# Sclerosing pneumocytoma diagnosed by preoperative endobronchial ultrasound-guided transbronchial needle aspiration (EBUS-TBNA)

**DOI:** 10.1186/s40792-018-0429-0

**Published:** 2018-03-09

**Authors:** Yuki Shiina, Yuichi Sakairi, Hironobu Wada, Hajime Tamura, Taiki Fujiwara, Takahiro Nakajima, Hidemi Suzuki, Masako Chiyo, Masayuki Ota, Satoshi Ota, Yukio Nakatani, Ichiro Yoshino

**Affiliations:** 1 Department of General Thoracic Surgery, Chiba University Graduate School of Medicine, 1-8-1, inohana, Chu-o-ku, Chiba, Chiba Japan; 2 Department of Diagnostic Pathology, Chiba University Graduate School of Medicine, 1-8-1, inohana, Chu-o-ku, Chiba, Chiba Japan

**Keywords:** Sclerosing pneumocytoma, EBUS-TBNA, Lung tumor

## Abstract

**Background:**

Sclerosing pneumocytoma is a rare lung tumor that is usually recognized as a solitary nodule in the lung. Surgical removal is recommended; however, its clinical diagnosis is still an issue because it is difficult to differentiate from lung adenocarcinomas using a tiny sample obtained from biopsy.

**Case presentation:**

We report a case of pulmonary sclerosing pneumocytoma located in the upper lobe of the right lung of a 34-year-old woman, which was diagnosed before surgery by endobronchial ultrasound-guided transbronchial needle aspiration (EBUS-TBNA).

A 3-cm irregular mass was detected by chest X-ray without any symptoms. She was referred to our hospital after being followed for 10 years in her previous clinic. During this follow-up period, the tumor had grown to 5 cm. We performed the EBUS-TBNA for the diagnosis. The histological findings obtained by EBUS-TBNA consisted of alveolar type 2-like cells that were positive for napsin A and round cells that were positive for vimentin. Based on these immunostaining results, we successfully diagnosed sclerosing pneumocytoma before surgery. Right upper lobectomy was performed, and the pathological diagnosis of the surgical specimen was also confirmed as sclerosing pneumocytoma.

**Conclusions:**

We herein report a case of sclerosing pneumocytoma, which was clinically diagnosed by EBUS-TBNA and resected surgically.

## Background

Sclerosing pneumocytoma is a rare and slow-growing benign tumor of the lung, and clinical diagnosis through biopsy is often difficult to differentiate from lung adenocarcinomas or carcinoids [1–3]. Preoperative biopsy samples, which are usually obtained as small fragments, are not adequate for definitive diagnosis of sclerosing pneumocytoma, which requires immunohistochemistry [4].

EBUS-TBNA is a minimally invasive method of hilar/mediastinal biopsy from parabronchial lesions that is mainly used for the diagnosis or nodal staging of lung cancer. EBUS-TBNA shows high accuracy and a low false-negative rate [5] and is also applied in the diagnosis of parabronchial neoplasms [6].

However, there are no reports which have so far described the efficacy of EBUS-TBNA in the diagnosis of sclerosing pneumocytoma. We experienced a case of sclerosing pneumocytoma that was diagnosed by preoperative EBUS-TBNA.

## Case presentation

**Fig. 1:**
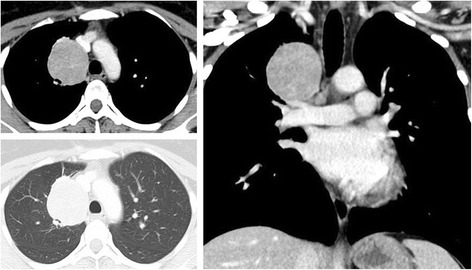
Contrast-enhanced chest tomography shows a 5.0 × 4.0 cm tumor in the right upper lobe. The tumor is adjacent to the right pulmonary artery and mediastinum

The patient was a 34-year-old asymptomatic woman who visited our hospital due to an abnormal nodule of the right lung that was detected on a chest X-ray and who had been followed up for 10 years in a previous clinic. Computerized tomography (CT) revealed that the tumor had grown from 3.0 to 5.0 cm during the 10-year follow-up period and that it was well enhanced and adjacent to the right pulmonary artery (Fig. 1). Fluorodeoxyglucose positron emission tomography (FDG-PET) demonstrated the increased uptake of FDG by the tumor (maximum standardized uptake value = 5.50); no other abnormal accumulation was observed. According to the clinical history and radiological findings, we suspected malignancy, including slow-growing lung carcinoma, or a mediastinal tumor, such as lymphoma or teratoma.

**Fig. 2:**
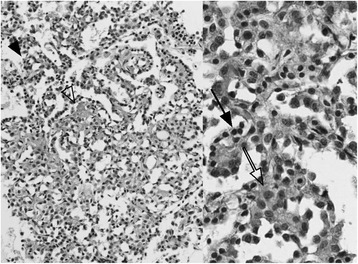
HE (hematoxylin-eosin) staining of a histological core specimen obtained by EBUS-TBNA. Left panel (low-power field): The tumor showed a papillary growth pattern (black arrowhead) with a mildly sclerotic stroma (outline arrowhead). Right panel (high-power field): The tumor consisted of peripheral cuboidal cells (black arrow) and oval stromal cells (outline arrow)

**Fig. 3:**
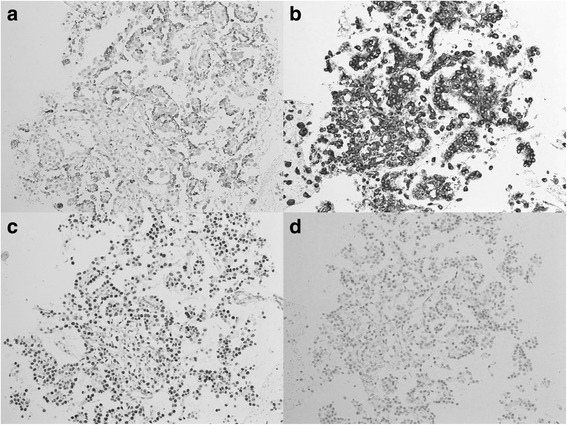
Immunohistochemical staining of the histological core specimen obtained by EBUS-TBNA. **a** The peripheral cells were positive for napsin A. **b** The oval stromal cells were positive for vimentin. **c** The biphasic cells were diffusely positive for TTF-1. **d** The biphasic cells were also partly positive for PgR

The patient underwent EBUS-TBNA under local anesthesia. EBUS-TBNA revealed hypervascular tumor adjacent to the trachea, and in total, three punctures were performed from different locations of tumor using 22-G needles; this obtained a sufficient amount of the histological core. No complications were observed. Histologically, the core showed a papillary growth pattern and consisted of peripheral cuboidal cells and oval stromal cells with mild nuclear atypia with low nuclear-cytoplasmic ratios (Fig. 2). The peripheral cells had an alveolar type 2 cell-like morphology, and the underlying stroma was mildly sclerotic. Immunostaining of the histological core (obtained by EBUS-TBNA) revealed three characteristic findings: (1) the biphasic cells were diffusely positive for TTF-1(thyroid transcription factor 1) and partly positive for PgR (progesterone receptor); (2) the peripheral cells were positive for napsin A; and (3) the stromal cells were positive for vimentin. Based on these findings, the tumor was finally diagnosed as sclerosing pneumocytoma (Fig. 3). The patient underwent right upper lobectomy and lymph node sampling. For complete resection with a secure margin, lobectomy was the minimal requirement due to CT findings of vascular invasion. The pathological diagnosis of surgical specimen was also sclerosing pneumocytoma, with no lymph node metastasis or vascular invasion. Postoperatively, the patient recovered well and has remained alive for 6 months without any recurrence after surgery.

### Discussion

Sclerosing pneumocytoma is a slow-growing benign tumor derived from lung alveolar cells that was previously known as sclerosing hemangioma. It was first reported by Liebow and Hubbell in 1956 [7]. This relatively rare tumor accounts for approximately 18% of benign lung tumors [8]. The tumor typically occurs asymptomatically in middle-aged women [4].

The CT and magnetic resonance imaging(MRI) features show extreme variation [9]. Clinically, it is difficult to reach a definitive diagnosis based on imaging alone. In this case, lung cancers and lymphoproliferative diseases were listed for differential diagnosis. Pathologically, the tumor is composed of solid, papillary, sclerotic, or hemangiomatous components. Regarding cell types, eosinophilic cuboidal epithelial cells may line either the papillary structures or slit-like spaces or sheets of round cells [10]. These pathological features are required for a definitive diagnosis of sclerosing pneumocytoma to differentiate from adenocarcinomas. It is usually difficult to diagnose using small specimens, such as those used for transbronchial cytological examination or intraoperative pathological examination. Iyoda et al. reported that only 7 of 26 sclerosing pneumocytoma patients were preoperatively diagnosed by percutaneous needle biopsy or transbronchial biopsy. Nine of the other 19 patients were intraoperatively diagnosed by touch smear cytology or the examination of frozen sections, and the remaining 10 patients were diagnosed based on the examination of surgical specimens [4]. These reports reveal that a specimen of sufficient size is required to make a correct preoperative or intraoperative diagnosis. EBUS-TBNA can obtain sufficient sample for histological examinations (including immunohistochemistry) under real-time guidance [11]. Obtaining a specimen of sufficient size (16 × 6 mm in this case) by EBUS-TBNA enables immunohistochemistry analysis, which is required for verification of pathologic diagnosis of sclerosing pneumocytoma.

Surgical resection is recommended for this disease, and several reports showed that similar surgical outcomes are obtained by lobectomy and sublobar resection [12]. We usually perform sublobar resection for sclerosing pneumocytoma; however, considering the tumor size and margin in this case, we considered lobectomy to be necessary for complete resection. In terms of the prognosis, a case with local recurrence at 10 years after sublobar resection was reported [13], and 2 of 24 patients with sclerosing pneumocytoma with lymph node involvement showed postoperative recurrence (in the lung parenchyma and vertebra, respectively) [14]. There is still no standard operation for sclerosing pneumocytoma; however, radical operation would be indicated if this disease is diagnosed.

## Conclusions

We were able to make an accurate preoperative diagnosis of sclerosing pneumocytoma based on the findings of a specimen obtained by EBUS-TBNA.

## Abbreviations

CT: Computerized tomography
EBUS-TBNA: Endobronchial ultrasound-guided transbronchial needle aspiration
FDG-PET: Fluorodeoxyglucose positron emission tomography
MRI: Magnetic resonance imaging
PgR: Progesterone receptor
TTF-1: Thyroid transcription factor
